# Genome sequencing and genetic characterization of Culex Flavirirus (CxFV) provides new information about its genotypes

**DOI:** 10.1186/s12985-016-0614-3

**Published:** 2016-09-23

**Authors:** Cíntia Bittar, Daiane Cristina Machado, Danila Vedovello, Leila Sabrina Ullmann, Paula Rahal, João Pessoa Araújo Junior, Maurício Lacerda Nogueira

**Affiliations:** 1 UNESP – São Paulo State University, Rua Cristóvão Colombo, 2265 – Jardim Nazareth, CEP 15054-000 São José do Rio Preto, SP Brazil; 2 FAMERP – Faculdade de Medicina de São José do Rio Preto, Av. Brigadeiro Faria Lima, 5416, Bloco U6 – Vila São Pedro, CEP: 15090-000 São José do Rio Preto, SP Brazil; 3 UNESP – São Paulo State University, R. Prof. Dr. Antônio Celso Wagner Zanin S/N Bairro: Distrito de Rubião Junior, Botucatu, SP CEP 18618-689 Brazil

**Keywords:** Culex flavivirus, Phylogeny, Genotypes

## Abstract

**Background:**

Culex Flavivirus (CxFV) is an insect-specific virus that is widely distributed and primarily infects mosquito species from the genus *Culex*. Its hosts include *Culex tritaeniorhynchus*, *Culex quinquefasciatus*, and *Anopheles sinensis* mosquitoes. Since its original identification, CxFV has been reported in several countries. Despite the increasing number of reports on CxFV, little is known about its genomic characteristics. It is unclear whether the phylogenetic relationships between the strains are influenced by host species and geographic location.

**Results:**

We characterized the Brazilian CxFV strain and performed a comprehensive genetic and phylogenetic characterization of CxFV based on all ORF sequences described so far. Our results revealed that the Brazilian strain is in a monophyletic clade with the Mexican strain. Overall, selective pressure indicates that the ORF is undergoing purifying selection.

**Conclusions:**

The phylogenetic analysis revealed a strong association between climate and CxFV ancestry. Also, based on phylogeny and the genetic distance between the main branches of the tree, we propose the classification of the available sequences into two different genotypes. We also suggest the existence of two different subtypes within Genotype 1.

**Electronic supplementary material:**

The online version of this article (doi:10.1186/s12985-016-0614-3) contains supplementary material, which is available to authorized users.

## Background

*Flaviviruses* are well known for causing important mosquito-borne human diseases. They are transmitted between arthropods and vertebrates, and are capable of replicating in both hosts. In 1975, a new member of this genus, incapable of replicating in vertebrate cells, was isolated from mosquito cell cultures and named Cell Fusing Agent Virus (CFAV) [1]. More recently, the isolation of other insect-specific viruses has been reported [2–5]. One of these insect-specific viruses is Culex Flavivirus (CxFV), an insect-specific virus that was first identified in 2007 in *Culex pipiens* mosquitoes [6]. Other hosts include *Culex tritaeniorhynchus*, *Culex quinquefasciatus*, and *Anopheles sinensis* mosquitoes [5, 7, 8].

Similar to other *flaviviruses*, CxFV encodes a polyprotein from a single-strand positive RNA open reading frame (ORF), flanked by 3′ and 5′-untranslated regions (UTR). The polyprotein of 3,364 aa is cleaved during and after translation into structural and non-structural proteins in the following order: C-prM(M)-E-NS1-NS2A-NS2B-NS3-NS4A-2K-NS4B-NS5 [6].

Since its identification, Culex Flavivirus has been reported in the United States, Mexico, Guatemala, Trinidad, Uganda, Indonesia, Japan, China, and Brazil, indicating that it is widely distributed [5, 6, 9–14]. Despite the increasing number of CxFV sequences deposited in GenBank, it is not clear if the phylogenetic relationships between the strains are influenced by the host species or geographic location. Moreover, little is known about the genetic profile of the CxFV genome.

Previously, we reported the first identification of CxFV in Brazil [14]. Here, we perform a genetic and phylogenetic analysis of the complete ORF of the Brazilian CxFV strain. In addition, we perform the genetic characterization of CxFV based on the ORF sequences described so far, and we propose a novel genotype classification.

## Methods

### Culex Flavivirus (CxFV) strain

According to report published previously by this research group [14], The CxFV strain obtained in this study was derived from *Culex quinquefasciatus* mosquitoes, collected between April 2007 and January 2008, in the city of São José do Rio Preto (SJRP). The mosquitoes were pooled according to genus, gender and geographic location, for RNA extraction using Trizol protocol (Invitrogen).

The presence of *Flavivirus* was detected for each pool by Multiplex-Nested_RT-PCR [15]. Ten positives pools were inoculated three times passages on C6/36. After the third passage pools were tested with primers specific for Culex Flavivirus (CxFV), Saint Louis Encephalitis Virus (SLEV), and West Nile Virus (WNV) [14]. One CxFV isolated was used for sequence analysis in this manuscript.

### Next generation sequencing

The CxFV RNA extracted from the C6/36 supernatant was quantified using a UV/Vis spectrophotometer (PicoDrop P200, Picodrop, Hinxton, Cambridgeshire, United Kingdom) and treated with Deoxyribonuclease (DNase I, Invitrogen, Carlsbad, CA, USA) to digest single and double-stranded DNA. cDNA was obtained using commercial SuperScript® III Reverse Transcriptase (Thermo Scientific, San Jose, CA, USA) according to the manufacturer’s protocol. A multiplex nested PCR was used once again to confirm CxFV positive sample [14, 15].

Double-stranded DNA was obtained using the SuperScript® Double-Stranded cDNA Synthesis Kit (Thermo Scientific, San Jose, CA, USA), purified with commercial magnetic beads (Agencourt AMPure XP, Beckman Coulter Inc., Indianapolis, IN, USA), and quantified. Sample were processed with the Nextera XT DNA Library Preparation Kit and sequenced using a commercial kit (MiSeq System, Illumina Inc., San Diego, CA, USA).

The assembly was performed by Geneious R6 (Biomatters, Auckland, New Zealand), using sequence of Toyama740/2005 CxFV strain (gb AB701772) as reference to assemble it.

### Culex flavivirus sequences

All the analyses in this study were based on a 26-sequence dataset composed of all complete Culex Flavivirus ORF sequences (10,092 nucleotides) deposited in GenBank at the time of the study, plus the Brazilian sequence described here (KT726939). Their accession numbers are: AB701776.1, AB701775.1, AB701774.1, AB701773.1, AB701772.1, AB701771.1, AB701770.1, AB701769.1, AB701768.1, AB701767.1, AB701766.1, JQ308190.1, JQ308189.1, JQ308188.1, JQ308187.1, JQ308186.1, HQ678513.1, FJ663034.1, GQ165808.1, FJ502995.1, NC_008604.2, JQ518484.1, EU879060.1, AB377213.1, and AB262759.2. All Sequences were aligned using Muscle in the SeaView 4.4.2 package [16, 17].

### Similarity analysis

Similarity analysis was carried out using the Sequence Identity Matrix tool from the BioEdit 7.0.5.3 package [18].

### Genetic distance

Mean genetic distances for the CxFV ORF and each specific region were calculated by the Kimura 2-parameters substitution model using Mega 6 [19].

The site-by-site genetic distances between CxFV_BR-RP01/2007 (KT726939) and the sequences identified in other countries were calculated in SimPlot 3.5.1 [20]. The analysis was carried out using the Kimura 2-parameter distance model and a 200-bp sliding window. Sequences were grouped by geographic location.

### Selective pressure analysis

Global and site-by-site ω were estimated in HyPhy [21]. Global ω was calculated for the full ORF and for each genomic region using the HKY85_3x4 substitution model and global parameters. Site-specific selection was calculated Single Likelihood Ancestor Counting (SLAC) method, the MG94xHKY85 model, and a *p*-value of 0.05.

As HyPhy requires a starting tree for all analysis, a maximum-likelihood tree was constructed for the entire ORF and for specific regions by PhyML in the SeaView package [22]. The GTR model was used, with optimized nucleotide equilibrium frequencies and site rate variation.

### Phylogenetic analysis

A Bayesian Markov Chain Monte Carlo (MCMC) analysis was used to estimate the phylogenetic tree using BEAST v1.8 [23]. The analysis was performed under strict molecular clock using as tree prior a constant population size coalescent and MCMC was run for 10,000,000 steps and sampled every 1000 steps. Trees were summarized using Tree Annotator 1.8 available on Beast package and edited using FigTree 1.4 [24]. BaTS 0.9 was used to statistically test for association between tree topology and geography, host species or climate [25].

The mean genetic distance between the sequences in specific branches of the phylogenetic tree was calculated in Mega 6 [19]. Sequences were divided into groups according to their distribution in the phylogenetic tree and the mean base substitution per site between groups was conducted using the Kimura 2-parameters substitution model [26].

## Results

### Culex Flavivirus (CxFV) identification

After collection, the mosquitoes were identified by gender and grouped into pools. A total of 83 pools of female *Culex quinquefasciatus*, ten mosquitoes per pool, were analyzed. Among the samples, 41 (49.4 %) were positive for CxFV in multiplex RT-PCR and ten were isolated in C6/36. After three passages in C6/36 cells isolation was confirmed from pool 73C by Multiplex-RT-PCR. The complete genome of the isolate from pool 73C was perform by Illumina Plataform.

### Next generation sequencing

NGS by Illumina generated a total of 69,242 reads of coverage, with 60,405 of CxFV reads, that generated 10,706 nucleotides. The sequence obtained was analyzed on BLAST, for confirmation of its contents and was deposited on GenBank under the accession number KT726939 and is here referred as CxFV_BR-RP01/2007.

### Similarity analysis

The sequence of the open reading frame (ORF) of the Brazilian strain was aligned with all available CxFV full genome sequences deposited on GenBank. The percentage of identity between the sequences was determined with the BioEdit package using CxFV_BR-RP01/2007 as the query sequence [18]. The highest similarity was observed between the Brazilian sequence and the sequence from Mexico (98.5 %) followed by that of Uganda (98.2 %) (Additional file 1: Table S1). All other sequences were approximately 90 % identical to CxFV_BR-RP01/2007.

### Genetic distance

**Fig. 1:**
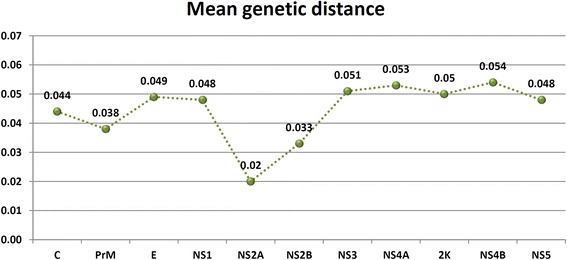
Overall mean genetic distance for CxFV specific genomic regions

Overall mean genetic distance was calculated for the complete ORF and for each specific genomic region. The ORF mean distance among all deposited sequences was 0.046. The mean genetic distance for each specific region is shown in Fig. 1. The NS2A region presented the lowest genetic distance and NS4B the highest.

**Fig. 2:**
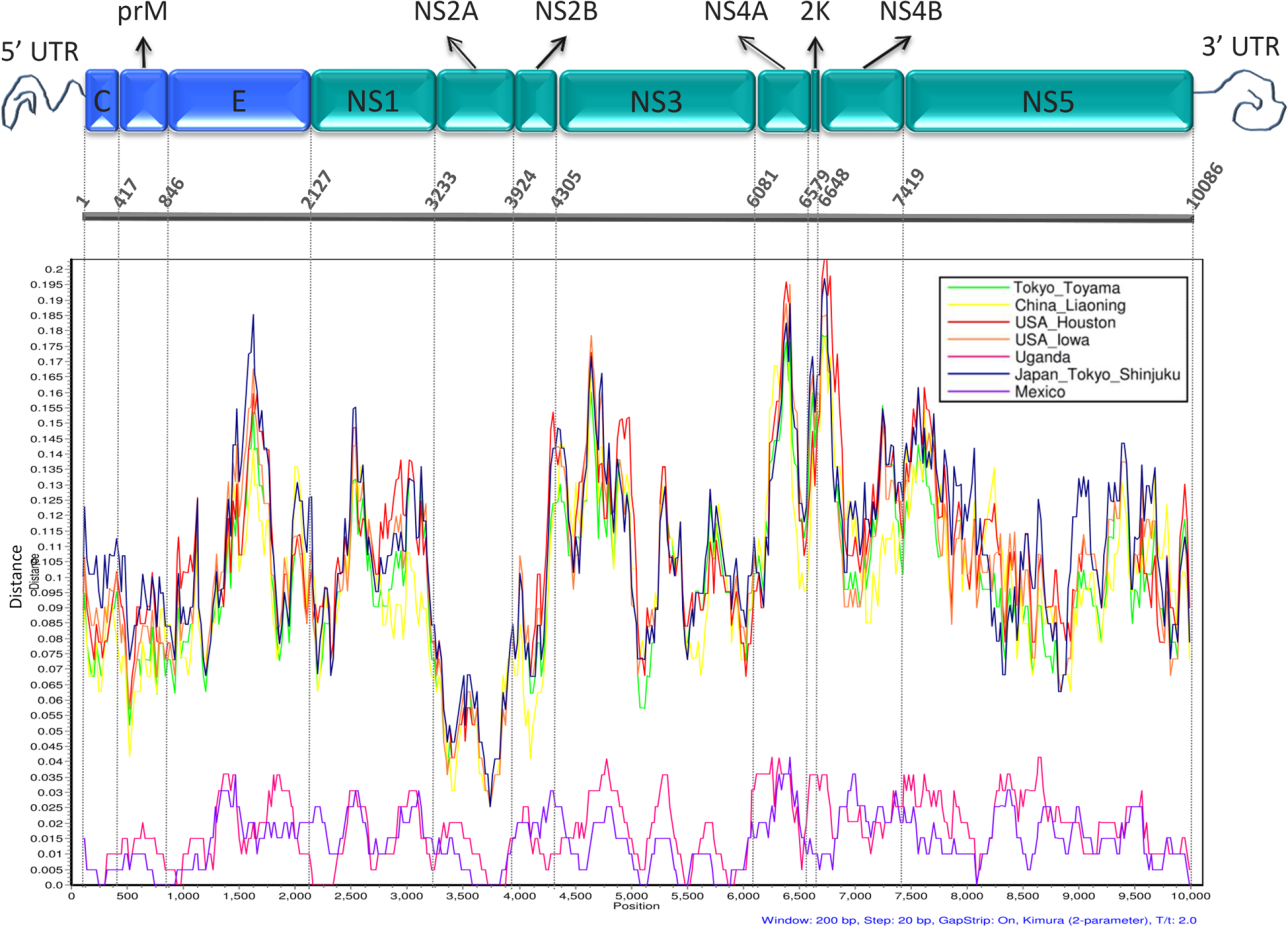
Genetic distance between the Brazilian sequence and isolates from other countries along the CxFV ORF. Analysis carried out on Simplot 3.5.1 with a 200 bp sliding window, Kimura 2-parameters distance model with CxFV_BR-RP01/2007 as query. Sequences were grouped by geographic location as specified in the legend. Schematic representation of CxFV genome based on the information provided by GenBank NC_008604.2. Structural proteins represented in *blue* and non-structural proteins represented in *green*

A site-by-site genetic distance analysis was performed on SimPlot in order to compare the Brazilian sequence with those from other countries. Sequences were grouped by geographic location. The resulting plot is shown in Fig. 2, which also depicts each genomic region. The plot reveals a lower genetic distance between the Brazilian and the Mexican and Ugandan isolates. All other sequences show similar genetic distances along the genome. The genomic region with the lowest genetic distance for all geographic locations is the NS2A region.

### Selective pressure analysis

**Fig. 3:**
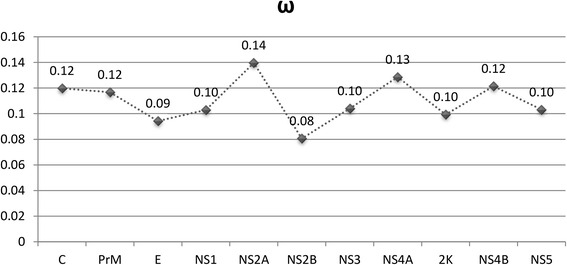
Global ω estimated for each genomic region separately using HyPhy

Global ω was estimated for the entire CxFV ORF and for each genomic region separately using HyPhy [21]. Our results indicate that the whole ORF is under strong purifying selection (ω = 0.107). The same applies for the individual genes encoding proteins as shown in Fig. 3. Interestingly, the NS2A region presented the highest ω (0.14), which is in contrast with the site-by-site genetic distance results, where this region presented the lowest values.

**Fig. 4:**
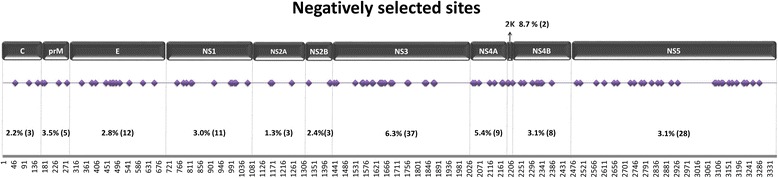
CxFV amino acid sites under negative selection. Percentage of negatively selected sites and (n) are indicated for each region. Schematic representation of CxFV genome based on the reference sequence NC_008604.2

Site-specific selection analysis revealed 106 negatively selected and no positively selected sites. Results are graphically represented in Fig. 4. NS3 presented more negatively selected sites (6.3 %). The NS2A (1.3 %) protein is the one with less negatively selected sites followed by Core (2.2 %).

### Phylogenetic analysis

**Fig. 5:**
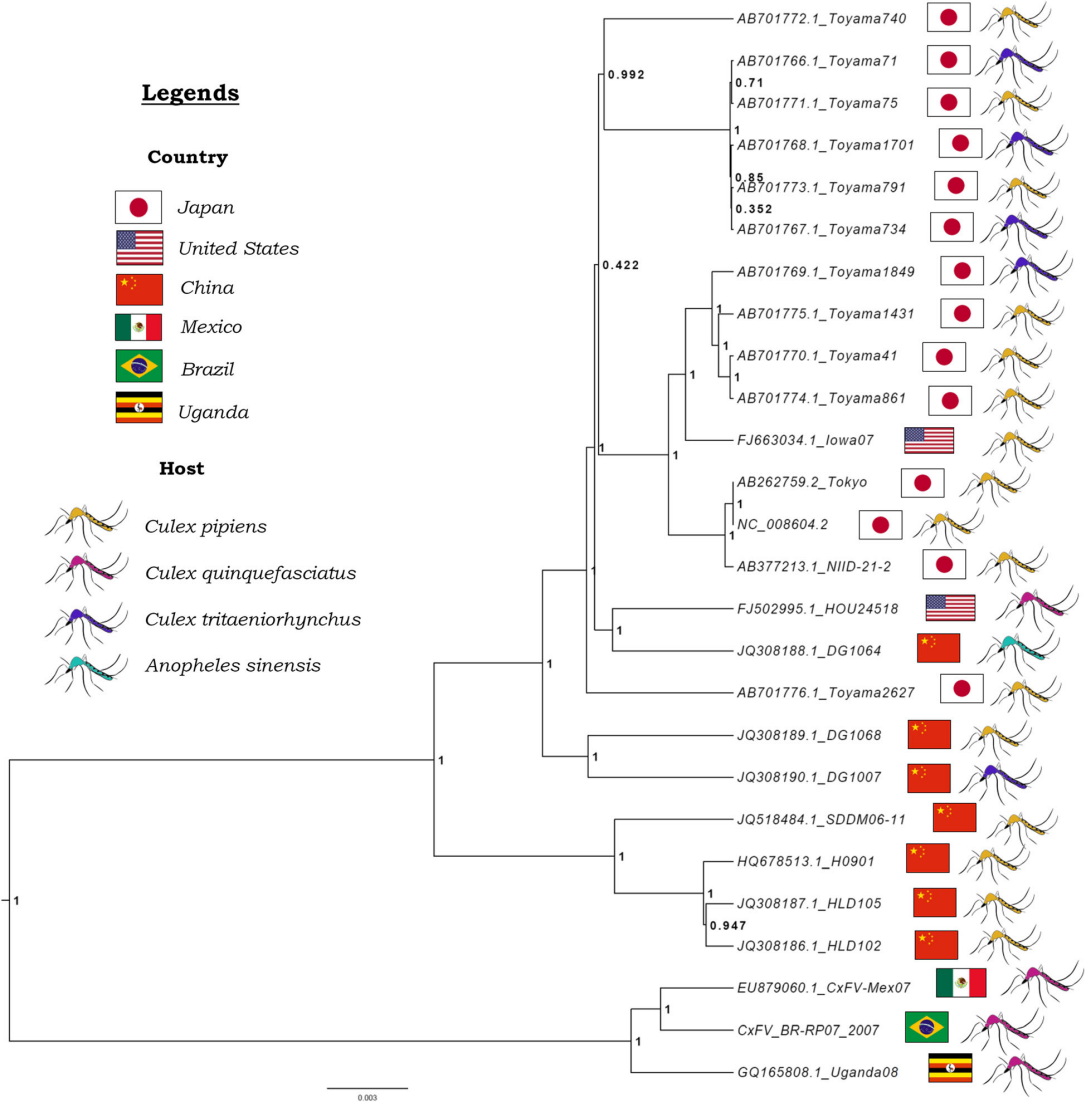
Bayesian phylogenetic tree of 26 sequences from CxFV ORF. Node labels represent the posterior probability. Country and host information is presented beside sequence name according to the legend above

The phylogenetic tree presented in Fig. 5 was reconstructed based on the 26 full ORF sequence. It is composed of two main monophyletic Clades with high confidence (Posterior probablity: 1). *Cade 1* includes most sequences, which are distributed in two groups, also supported high posterior probability (1). *Clade 2* comprises viruses from Brazil, Mexico, and Uganda. All members of this clade share the same host, *Culex quinquefasciatus*. However, this is the same host species as that of the strain collected in Houston, USA, which groups in *Clade 1*.

In order to test for association of phylogeny with either host, geographical location or climate the posterior set of trees estimated by BEAST were analyzed by BaTS, which quantifies and statistically tests the phylogeny-trait correlation. Evidence of strong association was detected between phylogeny and climate (*p* < 0.05). Also, Association index (AI) and parsimony score (PS) indicate association for both host and geographical location (*p* < 0.05). However monophyletic clade (MC) significance reveals that it only applies for Japan and China (location) and *Culex quinquefasciatus* (host) (Additional files 2–4: Tables S2–S4).

In order to estimate the evolutionary divergence between the main clades of the phylogenetic tree, the mean genetic distances between these groups were calculated. Our results show that *Clade 1* and *Clade 2* diverge 10.4 %, while the two main branches that compose *Clade 1* diverge 5.1 %.

## Discussion

Insect-specific flaviviruses, including CxFV, are a highly divergent group within the *Flavivirus* genus that shares a common ancestor with all other members, including the disease-causing ones. Understanding the genetic characteristics of CxFV is important not only to understand its biology and evolutionary history, but also to clarify some aspects of the whole genus.

Culex Flavivirus primarily infects globally distributed mosquito species of the genus *Culex*, which are vectors for pathogenic flaviviruses like WNV, SLEV, and JEV [27–29]. The most represented hosts in this work, *Culex pipiens* and *Culex quinquefasciatus,* are members of the *Culex pipiens* complex that consists of closely related species that are difficult to distinguish morphologically [30]. Studying the geographical distribution of the host is important to understand the evolutionary relationships between the identified CxFV strains. Two main monophyletic branches, *Clade 1* and *Clade 2*, can be observed in the phylogenetic tree with high branch support. Regarding countries, all Asian strains are grouped in *Clade 1* along with some sequences from USA. *Clade 2* is composed by sequences from different countries with only one sequence each. When considering host species, *Clade 2* is composed only of sequences derived from *Culex quinquefasciatus* while *Clade 1* as sequences from different hosts. This scenario prevents a clear association by simply analyzing the ancestry. Statistical analysis however revealed significant association between phylogeny and climate (temperate x tropical). *Clade 1* consists of virus from temperate climate regions while *Clade 2* is composed from sequences from region of tropical climate. Climate association is related with host species distribution. Although *Culex pipiens* occurs in temperate regions and *Culex quinquefasciatus* in tropical and subtropical regions their range overlaps and it has been proven they can hybridize [30–32]. When testing for host-phylogeny association only *Culex quinquefasciatus* was significantly associated. Moreover, country association was only found for Japan and China. Considering that both country and host are related to climate these factors may also be associated with CxFV ancestry, however the limited number of sequences from some hosts and geographic regions is probably preventing a more consistent conclusion.

We analyzed the genetic distance between the main branches of the phylogenetic tree that was reconstructed based on all CxFV ORF sequences described so far, including the Brazilian strain described in this work. *Clades 1* and *2* consist of strong groups with robust branch support, which are 10.4 % genetically distant. These observations sustain the idea that they could be two different genotypes of CxFV. For Japanese Encephalitis Virus, another *Culex* borne virus, genotypes are defined by 12 % difference in nucleotide composition of the highly divergent PrM gene [33, 34]. Genotypes of DENV, also a flavivirus, are defined by 6 % difference within a serotype based on the E region [35, 36]. In both cases, the limits were determined arbitrarily but supported by strong phylogenetic evidence such as ours. Other researchers have previously suggested that CxFV should be classified into different genotypes, based on the phylogenetic analysis of the E genomic region [37]. According to those authors, *Clade 1* described here would consist of Genotype 1 and *Clade 2* of Genotype 2. Our analysis also evidenced that the two main branches, with strong branch support (1000), in *Clade 1* diverge 5.1 %. These two branches could be considered different subtypes of CxFV Genotype 1 (1a and 1b). Similar results were obtained using the E region (Additional file 5: Table S5), suggesting it is a suitable region for CxFV genotyping and subtyping. We propose that groups of CxFV that are more than 9 % genetically distant could be classified as different genotypes and that differences higher than 4 % within these groups could be considered different subtypes. Although the cut-off values are arbitrarily defined, based on the genetic data available so far, they seem suitable.

Overall, selective pressure analysis indicates that the entire ORF, as well as specific regions, are undergoing purifying selection. Accordingly, site-by-site analysis found no positively selected sites and 106 sites under negative selection. This suggests that the virus is well adapted to its host and evolutionary forces are working against amino acid change. The infection of *Culex* mosquitoes by CxFV apparently does not result in disease. This observation is supported by the fact that the infection of C6/36 and AeAl-2, two *Aedes albopictus* cell lines, results only occasionally in moderate cytopathic effects [5, 6, 12]. If this is the case, any change in the genomic composition could disrupt the fine balance between what is advantageous for the virus and at the same time does not negatively affect the host, supporting the purifying selection.

Although the differences are not substantial, the NS2A region presents higher ω values and lower percentages of negatively selected sites. It is interesting, however, to notice that the same region also showed the lowest genetic distance. At first, these data might seem in disagreement. However, it suggests that NS2A displays low genetic variability in general, but a more relaxed pressure against amino acid change when compared to other regions. CxFV and other *Flaviviruses* from the insect-specific group present a frameshift signal that produces an alternate reading frame called *fifo* [38]. The signal consists of a stem-loop structure in the RNA genome that is harbored inside the NS2A region [39]. In this case, the selection is acting not only on the protein, but also on the RNA structure to avoid disturbing the frameshift signal. It is important to highlight that different amino acids might have similar chemical properties and a change would not necessarily disrupt the tertiary structure. This could explain why, despite the low genetic distance, this region is more permissive to amino acid change.

## Conclusion

There are still many aspects of Culex Flavivirus and other insect-specific *flaviviruses* that needs to be clarified. *Culex ssp.* mosquitoes are distributed worldwide suggesting that CxFV should be present in more places than currently reported. Here we report an association between the ancestry of CxFV and climate. The identification of new strains from different countries and hosts is important for elucidating the ecology and evolutionary history of this virus. Based on the available sequences, we propose the classification of Culex Flavivirus into two genotypes (1 and 2) and the existence of two subtypes within genotype 1.

## Additional file

Additional file 1: Table S1. Percentage of identity between CxFV_BR-RP01/2007 and sequences from GenBank. Data determined by Sequence Identity Matrix tool on BioEdit package. **Table S2.** Results from BaTS when testing for country association. State 0 – Japan; State 1 – Brazil; State 2 – Mexico; State 3 – USA; State 4 – Uganda; State 5 – China; AI – Association Index; PS – Parsimony Score; MC – Monophyletic clade. **Table S3.** Results from BaTS when testing forhostassociation. State 0 – Culexpipiens; State 1 – Culextritaeniorhynchus; State 2 – Culexquinquefasciatus; State 3 – Anopheles sinensis; AI – Association Index; PS – Parsimony Score; MC – Monophyletic clade. **Table S4.** Results from BaTS when testing for climate association. State 0 – Temperate; State 1 – Tropical; AI – Association Index; PS – Parsimony Score; MC – Monophyletic clade. **Table S5.** Mean genetic distance between the sequences from envelope E region. Sequences were divided into groups according to their distribution in the phylogenetic tree and the mean base substitution per site between groups was conducted using the Kimura 2-parameters substitution model. Cluster 1 - KT726939, GQ165808.1, EU879060.1; Cluster 2 - JQ308187.1, JQ308186.1, JQ518484.1, HQ678513.1; Cluster 3 – All others. (DOCX 83.3 kb)
