# Supplementary material for: Genome sequencing and genetic characterization of Culex Flavirirus (CxFV) provides new information about its genotypes
Source: Virol J. 2016 Sep 23;13:158. doi: 10.1186/s12985-016-0614-3 (PMC5034531; doi:10.1186/s12985-016-0614-3)
Supplement: Additional file 1: Table S1. — Percentage of identity between CxFV_BR-RP01/2007 and sequences from GenBank. Data determined by Sequence Identity Matrix tool on BioEdit package. Table S2. Results from BaTS when testing for country association. State 0 – Japan; State 1 – Brazil; State 2 – Mexico; State 3 – USA; State 4 – Uganda; State 5 – China; AI – Association Index; PS – Parsimony Score; MC – Monophyletic clade. Table S3. Results from BaTS when testing forhostassociation. State 0 – Culexpipiens; State 1 – Culextritaeniorhynchus; State 2 – Culexquinquefasciatus; State 3 – Anopheles sinensis; AI – Association Index; PS – Parsimony Score; MC – Monophyletic clade. Table S4. Results from BaTS when testing for climate association. State 0 – Temperate; State 1 – Tropical; AI – Association Index; PS – Parsimony Score; MC – Monophyletic clade. Table S5. Mean genetic distance between the sequences from envelope E region. Sequences were divided into groups according to their distribution in the phylogenetic tree and the mean base substitution per site between groups was conducted using the Kimura 2-parameters substitution model. Cluster 1 - KT726939, GQ165808.1, EU879060.1; Cluster 2 - JQ308187.1, JQ308186.1, JQ518484.1, HQ678513.1; Cluster 3 – All others. (DOCX 83.3 kb) [file 12985_2016_614_MOESM1_ESM.docx]

# Supplementary Material

| **Sequence** | **CxFV_BR-RP01/2007** |
| --- | --- |
| EU879060.1_CxFV-Mex07 | 98.5% |
| GQ165808.1_Uganda08 | 98.2% |
| JQ308190.1_DG1007 | 90.5% |
| AB701772.1_Toyama740 | 90.4% |
| AB701776.1_Toyama2627 | 90.3% |
| AB701775.1_Toyama1431 | 90.3% |
| AB701774.1_Toyama861 | 90.3% |
| AB701773.1_Toyama791 | 90.3% |
| AB701771.1_Toyama75 | 90.3% |
| AB701770.1_Toyama41 | 90.3% |
| AB701769.1_Toyama1849 | 90.3% |
| AB701768.1_Toyama1701 | 90.3% |
| AB701767.1_Toyama734 | 90.3% |
| AB701766.1_Toyama71 | 90.3% |
| JQ308189.1_DG1068 | 90.3% |
| FJ663034.1_Iowa07 | 90.3% |
| HQ678513.1_H0901 | 90.2% |
| JQ308188.1_DG1064 | 90.1% |
| JQ308187.1_HLD105 | 90.1% |
| JQ308186.1_HLD102 | 90.1% |
| FJ502995.1_HOU24518 | 90.1% |
| JQ518484.1_SDDM06-11 | 90.1% |
| AB377213.1_NIID-21-2 | 90.0% |
| NC_008604.2 | 89.9% |
| AB262759.2_Tokyo | 89.9% |

**Table S1:** Percentage of identity between CxFV_BR-RP01/2007 and sequences from GenBank. Data determined by Sequence Identity Matrix tool on BioEdit package.


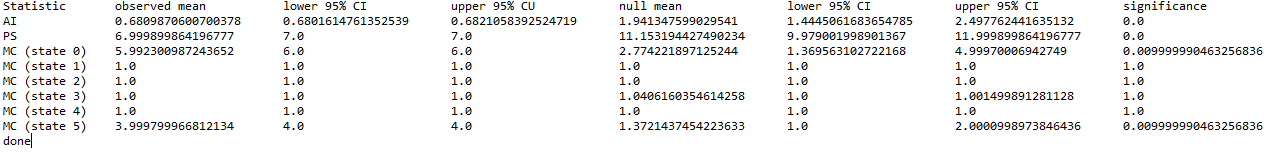


**Table S2:** Results from BaTS when testing for country association. State 0 – Japan; State 1 – Brazil; State 2 – Mexico; State 3 – USA; State 4 – Uganda; State 5 – China; AI – Association Index; PS – Parsimony Score; MC – Monophyletic clade.


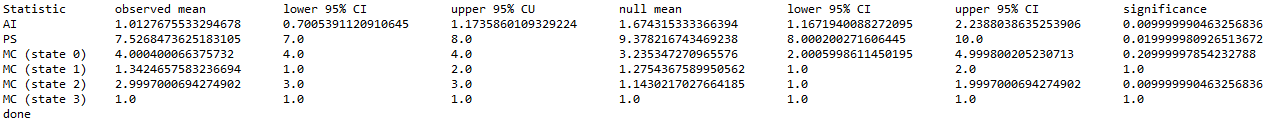

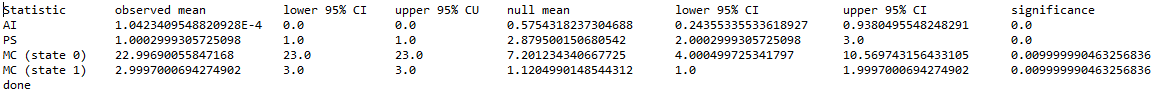


**Table S4:** Results from BaTS when testing for climate association. State 0 – Temperate; State 1 – Tropical; AI – Association Index; PS – Parsimony Score; MC – Monophyletic clade.

**Table S3:** Results from BaTS when testing for host association. State 0 – Culex pipiens; State 1 – Culex tritaeniorhynchus; State 2 – Culex quinquefasciatus; State 3 – Anopheles sinensis; AI – Association Index; PS – Parsimony Score; MC – Monophyletic clade.


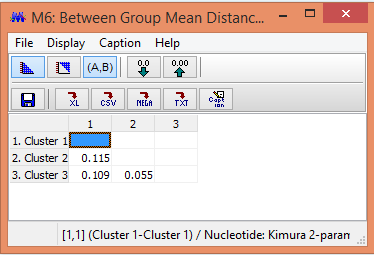


**Table S5:** Mean genetic distance between the sequences from envelope E region. Sequences were divided into groups according to their distribution in the phylogenetic tree and the mean base substitution per site between groups was conducted using the Kimura 2-parameters substitution model. Cluster 1 - KT726939, GQ165808.1, EU879060.1; Cluster 2 - JQ308187.1, JQ308186.1, JQ518484.1, HQ678513.1; Cluster 3 – All others.
